# The Influence of Physical Training on Breast Cancer: The Role of Exercise-Induced Myokines in Regulating Breast Cancer Cell Growth and Survival

**DOI:** 10.3390/ijms252111379

**Published:** 2024-10-23

**Authors:** Anirudh Natarajan, Rashmita Pradhan, Walburga Dieterich, Raphaela Schwappacher, Dejan Reljic, Hans J. Herrmann, Markus F. Neurath, Carolin C. Hack, Matthias W. Beckmann, Yurdagül Zopf

**Affiliations:** 1 Department of Medicine 1, Friedrich-Alexander-University Erlangen-Nürnberg, 91054 Erlangen, Germany; anirudh.natarajan@uk-erlangen.de (A.N.); rashmita.pradhan@uk-erlangen.de (R.P.); walburga.dieterich@uk-erlangen.de (W.D.); dejan.reljic@uk-erlangen.de (D.R.); hans.herrmann@uk-erlangen.de (H.J.H.); markus.neurath@uk-erlangen.de (M.F.N.); 2 Hector-Center for Nutrition, Exercise and Sports, Department of Medicine 1, Friedrich-Alexander-University Erlangen-Nürnberg, 91054 Erlangen, Germany; 3 Department of Gynaecology and Obstetrics, Comprehensive Cancer Center Erlangen-EMN (CCC ER-EMN), Universitätsklinikum Erlangen, Friedrich-Alexander-University Erlangen-Nürnberg, 91054 Erlangen, Germany; carolin.hack@uk-erlangen.de (C.C.H.); fk-direktion@uk-erlangen.de (M.W.B.)

**Keywords:** breast cancer, resistance training, whole-body electromyostimulation, electric pulse stimulation, myokines, proliferation, apoptosis

## Abstract

The beneficial impact of physical training in lowering cancer risk is well known. However, the precise mechanisms linking physical training and cancer are not fully understood. Skeletal muscle releases various myokines that seem to possess a direct anti-tumor effect. Although breast cancer (BC) is the prevalent form of cancer among women on a global scale, only limited data are available about the secretion of myokines following exercise in patients with BC. To study the effects of exercise on BC, the blood samples of patients with varied stages of BC were analyzed after 12 weeks of resistance training with whole-body electromyostimulation (WB-EMS). Following the training period, we observed that resistance training helps these patients to improve their physical characteristics and performance function by increasing skeletal muscle mass and strengthening their hand grip. Notably, the patient’s serum was found to inhibit the growth and promote the apoptosis of BC cells in vitro. Moreover, the conditioned medium collected from in vitro stimulated human myotubes using electric pulse stimulation (EPS), an in vitro simulation of WB-EMS training, induced the cell death of BC cells. These results highlighted the direct cancer-protective effects of activated skeletal muscle. In line with our observed effects of serum from exercise-trained pancreatic and prostate cancer patients, the growth of BC cells was notably inhibited when supplemented directly with recombinant myokines C-X-C motif ligand 1 (CXCL1), Interleukin 10 (IL10), and C-C motif chemokine ligand 4 (CCL4). Notably, treatment with these myokines also increased the expression of caspase 3/7 (*Casp3/7*), resulting in enhanced BC cell death. Our data strongly suggest that physical exercise has a positive impact on skeletal muscle mass and hand grip strength in BC patients, along with a significant anti-tumor effect in BC cells. This shows promising potential for considering sports and physical training as supportive therapies for achieving more impactful cancer treatment.

## 1. Introduction

Epidemiological investigations and clinical studies strongly indicate that regular physical exercise correlates with reduced morbidity and mortality rates among cancer patients [1,2]. However, the crosstalk between exercise and cancer is not fully understood. It has been shown that stimulated skeletal muscle releases myokines, a group of cytokines, that have the significant potential to inhibit the growth and viability of cancer cells by inducing apoptosis and enhancing immune function [3,4,5]. For example, a study utilizing isolated hind limbs from Wistar rats, which were electrically stimulated ex vivo to induce muscle contraction, demonstrated that the resulting myokine-rich conditioned medium could suppress the growth of breast cancer (BC) cells in vitro as well as in an in vivo rodent breast cancer model [6]. Identifying specific myokines responsible for this effect has significantly increased our understanding of the influence of muscle cells on the immune system and the possible inhibition of cancer cell growth. Apart from in vivo studies in murine models, the direct anti-cancer effects of exercise in humans have been little studied. The influence of exercise and sports on cancer in physically weakened patients or those undergoing chemotherapy and radiotherapy has hardly been investigated. Our research group showed that even patients in advanced cancer stages, who are physically very limited and under cancer-specific therapy, can build muscle with innovative training concepts and show increased myokine expression [7]. We demonstrated that resistance training with whole-body electromyostimulation (WB-EMS) impacts the growth and viability of human cancer cells. We were able to show that the serum of exercise-trained patients with advanced-stage pancreatic cancer (PC) leads to the release of certain myokines, such as C-X-C motif ligand 1 (CXCL1), Interleukin 10 (IL10), and C-C motif chemokine ligand 4 (CCL4), from contracting skeletal muscle. These specific myokines reduced cell proliferation and migration rates in these cancer cells in vitro. Thus, the findings indicate that stimulating muscle through resistance training (WB-EMS) can help control the expression of genes that regulate the growth and viability of cancer cells [4].

Over recent years, research in the field of BC has gained significant prominence, as it is the most common cancer in women globally. Studies are focused on individualized medicine and novel therapeutic approaches in this area. Physically active BC patients demonstrate better survival and a lower likelihood of recurrence [8]. Performing physical exercise can help mitigate the side effects of cancer treatment, such as fatigue, anxiety, and depression. It can also help to improve the overall quality of life. However, the fundamental molecular pathways still need to be understood. Thus, the present study aims to determine the importance of muscle gain and investigate the effects of myokine expression on proliferation and apoptosis in BC. Our data will provide further insights into the relationship between physical training and BC, the results of which may further improve individualized therapy.

## 2. Results

### 2.1. Study Design and Patient Data

There were no major differences between the two study groups in terms of Karnofsky index (performance status), six-minute walking distance (physical functioning), body mass index, skeletal muscle mass, and the routine blood parameters measured at the baseline (Table 1).

**Figure 1:**
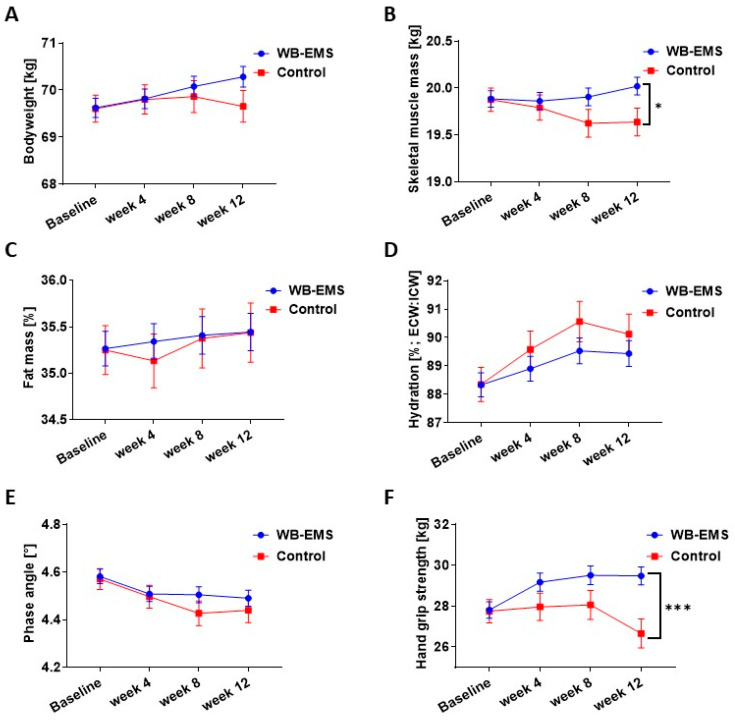
**Intervention data on the performance status, physical function, and body parameters of the two study groups** (Control group, n = 22, who completed the study; Resistance training (WB-EMS) group, n = 56, who completed the study). The mean values (standard error of the mean, SEM) of all patients in each study group are shown at baseline and weeks 4, 8, and 12, to visualize the trends in the parameters during the whole study period. (**A**–**E**): Body weight, skeletal muscle mass, fat mass, hydration (Extracellular water (ECW): Intracellular water (ICW)), and phase angle were determined by bioelectrical impedance analysis (Seca, Medical body composition analyzer). (**F**): Hand grip strength was measured with a Saehan hand dynamometer. Graphs display mean (SEM) values. Statistical analysis was performed using *t*-test, Student’s *t*-test, Wilcoxon signed-rank test, and Mann–Whitney test. * *p* < 0.05; *** *p* < 0.001.

After a 12-week intervention period, the resistance training (WB-EMS) group exhibited significant improvements compared to the control group. Specifically, body weight (Figure 1A), skeletal muscle mass (SMM; Figure 1B), and hand grip strength (Figure 1F) increased in the training group compared to the control group without training. Notably, the difference between the pre- and post-intervention measurements of SMM (0.38 ± 0.34 kg, *p* = 0.03) and hand grip strength (2.83 ± 1.75 kg, *p* = 0.001) exhibited a statistically significant improvement in response to physical training at week 12 compared to the control group (Supplementary Table S1). Moreover, hydration plays a role in managing cancer patients, with direct implications for improving muscle mass. Our results demonstrated an improved hydration level that was indicated by a reduced extracellular to intracellular water ratio, meaning that the proportion of water found outside the cells (extracellular) decreased relative to the water found inside the cells (intracellular) in patients undergoing physical training compared to the control group cohort during the 12 weeks (Figure 1D). We also determined the phase angle, which is associated with muscle mass and lean mass. A higher phase angle stands for an improved muscle mass. At the beginning of the study, a slight drop in the phase angle was observed, which then stabilized over the further course in patients who performed physical training (PhA; Figure 1E). Fat Mass (FM; Figure 1C) stayed considerably stable in the exercising group until the end of the study period; however, this parameter showed a slight decrease at week 4 in the control group, but came to a steady and stable level that paralleled the exercising group from there until the end. Also, significant differences were observed between the two study groups in the six-minute walking distance test when comparing pre- and post-intervention results (*p* = 0.0025) and within the resistance-trained group before and after the 12 weeks of intervention (*p* = 0.0007). Similarly, creatinine level measurements showed a significant effect (*p* = 0.041) when comparing pre- versus post-intervention results between physically trained patients and the control group (Supplementary Table S2). However, the other clinical parameters, including C-reactive protein level and Karnofsky index between the pre- and post-intervention, showed no significant result in both groups.

### 2.2. Exercise-Based Stimuli Affect BC Cell Proliferation and Apoptosis

**Figure 2:**
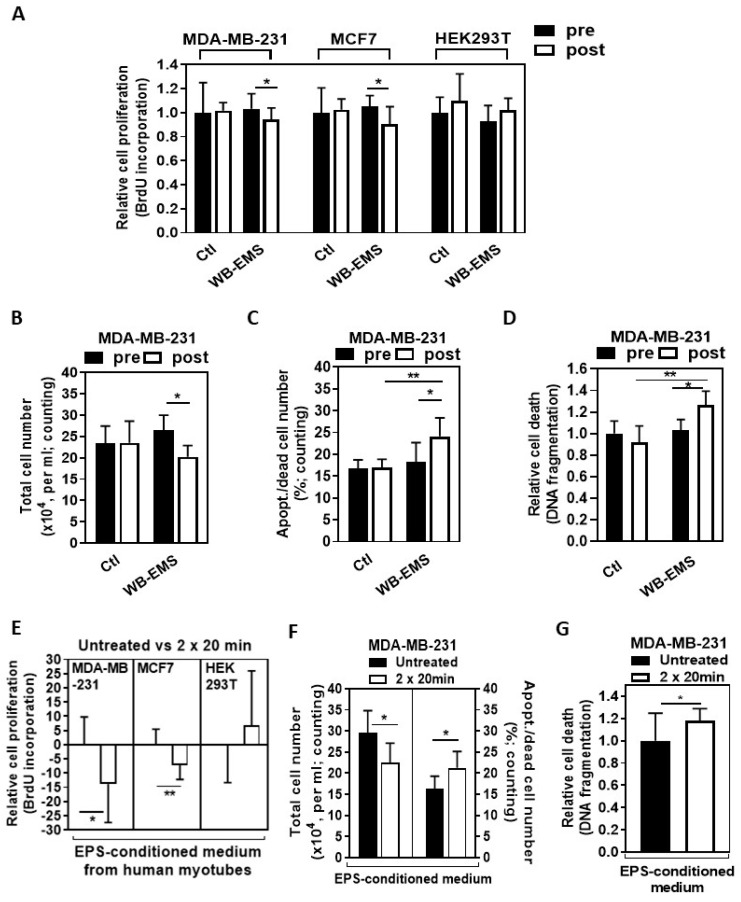
**Serum of patients with physical training (WB-EMS) and Electrical Pulse Stimulation (EPS)-conditioned medium from human myotubes influence the proliferation and apoptosis of BC cells.** (**A**). Cell lines MDA-MB-231 and Michigan Cancer Foundation-7 (MCF7), along with non-malignant Human Embryonic Kidney 293T (HEK293T) cells, were serum-starved in a medium containing 0.1% Fetal calf serum (FCS) for 18–24 h. Cells were then stimulated for 96 h with 10% serum collected from BC patients, taken either at the start of the study (pre) or after 12 weeks (post) of WB-EMS resistance training (control group (Ctl); physical training: WB-EMS). Cell proliferation was assessed and is presented relative to the values obtained after stimulation with a pre-intervention serum pool from control patients (n = 18). The number of independent biological replicates: MDA-MB-231 (n = 4), MCF7 (n = 5), and HEK293T (n = 4). (**B**,**C**). Starved MDA-MB-231 cells were treated with serum pools from WB-EMS or control BC patients. After 96 h, the number of viable total cells and the number of apoptotic cells were determined: (**B**) shown as total cell count of 1 × 10^4^; the number of independent biological replicates, n = 7, and (**C**) as number of apoptotic/dead cells; % of total cell number; the number of independent biological replicates (n = 7). (**D**). Apoptotic DeoxyriboNucleic acid (DNA) fragmentation in MDA-MB-231 cells was assessed after 48 h of treatment with WB-EMS-trained serum; the number of independent biological replicates was n = 6. (**E**). BC cells (MDA-MB-231, MCF7) and non-malignant cells (HEK293T) were serum-starved and treated with EPS-conditioned medium from in vitro differentiated human myotubes for 48 h. Cell proliferation was measured using the 5-bromo-2′-deoxyuridine (BrdU) assay. The graph shows the relative proliferation of untreated cells as 0%, with the percentage difference in proliferation after treatment with EPS-conditioned medium compared to the unconditioned medium; the number of independent biological replicates, n ≥ 6. (**F**). The effects of EPS-conditioned human myotube medium on the total cell count and the number of apoptotic/dead MDA-MB-231 cells assessed after 48 h; the number of independent biological replicates, n = 9. (**G**). Relative cell death of MDA-MB-231 after being incubated with EPS-conditioned human myotube medium for 48 h was measured; the number of independent biological replicates, n = 3. Graphs display mean (SD) values. Statistical analysis was carried out using a two-tailed *t*-test and Student’s *t*-test in (**A**,**D**,**E**,**G**); Wilcoxon signed-rank and Mann–Whitney test in (**B**,**C**,**F**). * *p* < 0.05, ** *p* < 0.01. minutes, min.

Sera from patients who performed WB-EMS resistance training significantly decreased cell proliferation of both BC cell lines (MDA-MB-231 and Michigan Cancer Foundation-7, MCF7), while the proliferation of non-malignant Human Embryonic Kidney 293T (HEK293T) cells was unaffected by these sera after a 96 h stimulation period (Figure 2A: MDA-MB-231, *p* < 0.05; MCF7, *p* < 0.05). The 96 h of incubation with the serum of patients with resistance training also significantly decreased the total number of MDA-MB-231 cells (Figure 2B: *p* < 0.05) and caused a significant increase in apoptotic MDA-MB-231 cells (Figure 2C: WB-EMS post versus Ctrl post, *p* < 0.01; WB-EMS post versus pre, *p* < 0.05). In addition, there was a highly significant increase in relative cell death (Figure 2D: WB-EMS post versus Ctrl post, *p* < 0.01; WB-EMS post versus pre, *p* < 0.05) when MDA-MB-231 cells were stimulated for 48 h with WB-EMS trained patient serum.

The EPS-conditioned myotube medium also significantly decreased the proliferation of the human BC cells (Figure 2E: 2 × 20 min EPS MDA-MB-231 versus untreated MDA-MB-231, *p* < 0.05; 2 × 20 min EPS MCF7 versus untreated MCF7, *p* < 0.01) while not inhibiting the non-malignant HEK293T cells after 48 h stimulation. In addition, while the total MDA-MB-231 cell number was significantly decreased (Figure 2F: *p* < 0.05), the apoptotic MDA-MB-231 cell number (Figure 2F; *p* < 0.05), and the relative MDA-MB-231 cell death (based on DNA fragmentation) increased significantly (Figure 2G: *p* < 0.05) after 48 h stimulation with the EPS-conditioned medium.

### 2.3. Exercise-Induced Myokines Mix (CXCL1, IL10, and CCL4) Control BC Cell Growth and Apoptosis

**Figure 3:**
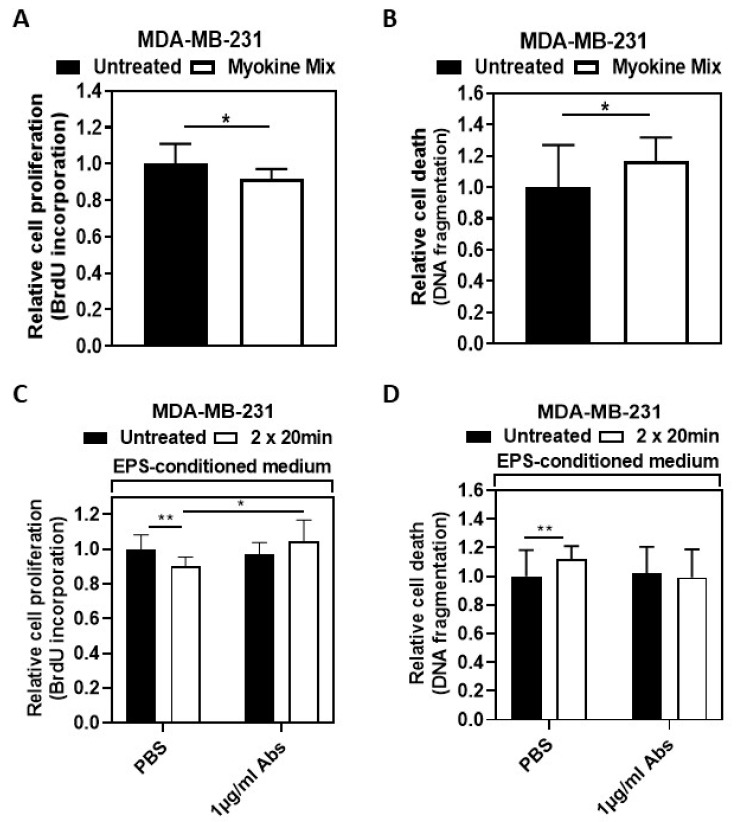
**Myokines CXCL1, IL10, and MIP-1β/CCL4 regulate BC cell proliferation and apoptosis.** MDA-MB-231 breast cancer cells were serum-starved in a medium containing 0.1% FCS for 18–24 h. (**A**). Then, the cells were treated with 20 ng/mL each of CXCL1, IL10, and MIP-1β/CCL4 for 48 h, followed by the measurement of BrdU uptake. The cytokine mixture significantly reduced cell proliferation compared to untreated cells, *p* < 0.05; independent biological replicates (n = 7). (**B**). DNA fragmentation in MDA-MB-231 cells was significantly evaluated after 48 h of treatment with the myokine mix, *p* < 0.05; independent replicates, n = 7. (**C**,**D**). Neutralization assay: Cell proliferation and DNA fragmentation in BC cells MDA-MB-231 were determined after neutralizing the effects of EPS-conditioned medium with specific counteracting antibodies for CXCL1, IL10, and CCL4/macrophage inflammatory protein-1 beta (MIP-1β) for 48 h. The antibodies (Abs) were used at concentrations of 1 µg/mL each. (**C**). Determination of cell proliferation by BrdU uptake measurement. The graph displays the reduced proliferation of cells incubated with EPS-conditioned medium (white bars) relative to untreated cells (black bars, set at 1.0) and the neutralizing effect of blocking antibodies. The number of independent biological replicates; n = 5. (**D**). Apoptotic DNA fragmentation measurement. The number of independent biological replicates, n = 7. Graphs display mean (SD) values. Statistical analysis was done using Student’s *t*-test in (**A**–**D**). * *p* < 0.05, ** *p* < 0.01. Phosphate-buffered saline, PBS.

The stimulation of MDA-MB-231 cells with the myokine mix of CXCL1, IL10, and CCL4/ macrophage inflammatory protein-1 beta (MIP-1β) for 48 h reduced the MDA-MB-231 cell proliferation significantly (Figure 3A: Myokine mix versus Untreated, *p* < 0.05) while causing a significant increase in the relative cell death (Figure 3B: *p* < 0.05).

In Figure 3C, the left-hand columns show the significant inhibitory effect of the EPS-conditioned medium on MDA-MB-231 cell proliferation (** *p* < 0.01). This effect was counteracted by incubating MDA-MB-231 cells with neutralizing antibodies for CXCX1, IL10, and MIP-1β. The incubation of MDA-MB-231 cells with the combination of 1 µg/mL of each blocking antibody resulted in a significantly increased proliferation rate of those cells that were incubated with the EPS-conditioned medium (Figure 3C, right-hand columns; * *p* < 0.05). However, the incubation with these blocking antibodies in addition to the EPS-conditioned medium showed no significant effect on the DNA fragmentation of MDA-MB-231 cells (Figure 3D).

### 2.4. Casp 3/7 mRNA Expression and Casp3/7 Activity as a Measure for Increased Apoptosis

**Figure 4:**
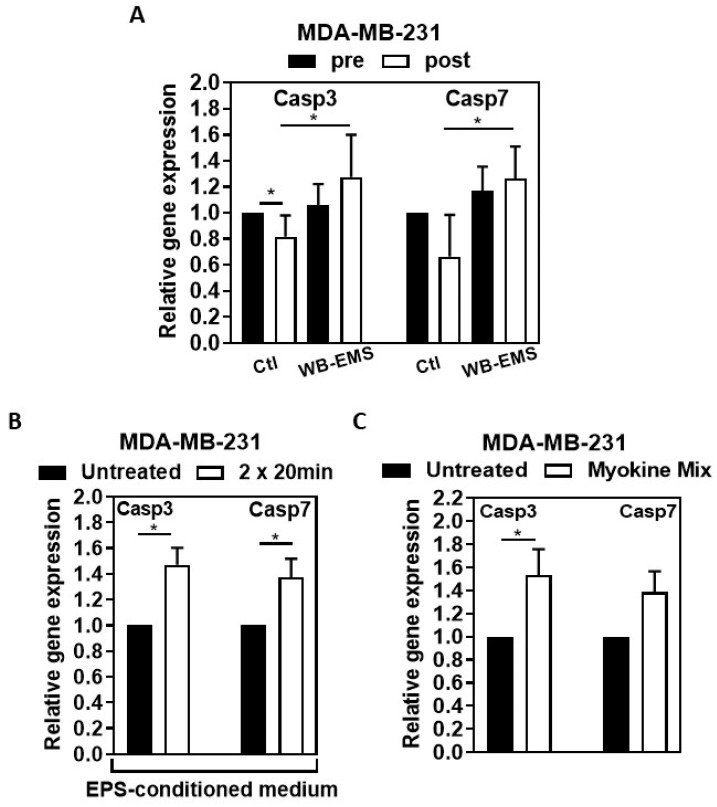
**WB-EMS trained patient serum, EPS-conditioned medium, and myokine mix increase the total Caspase 3 and 7 gene expression.** MDA-MB-231 cells were serum-starved with 0.1% FCS for 18–24 h and afterward stimulated either with: (**A**). serum from controls or WB-EMS trained BC patients, the number of independent biological replicates (n ≥ 5), (**B**). EPS-conditioned medium from human myotubes, the number of independent biological replicates (n = 4), or (**C**). with a combination of CXCL1, IL10, and CCL4 (20 ng/mL each), the number of independent biological replicates, n ≥ 6 for 72 h. Then, caspase 3 and 7 messenger RiboNucleic Acid (mRNA) expression levels were analyzed, and results were depicted in the graph as fold changes relative to the respective control/untreated samples. Untreated cells were set at 1.0. Graphs display mean (SD) values. Statistical analysis was performed using two-tailed *t*-test in A and Student’s *t*-test in (**B**,**C**). * *p* < 0.05.

A significant decrease in *Casp3* was observed when MDA-MB-231 breast cancer cells were incubated with post-intervention sera of control patients without resistance training. In addition, an apparent reduction in *Casp7* was determined in MDA-MB-231 cells after incubation with the post-intervention sera of these controls, although reaching no significance. However, sera from patients performing 12 weeks of WB-EMS training resulted in significantly upregulated *Casp3* and *Casp7* gene expression in MDA-MB-231 cells compared to the sera of the control group, *p* < 0.05 (Figure 4A). A significant upregulation of the *Casp3* and *Casp7* genes was also achieved when MDA-MB-231 cells were stimulated with the EPS-conditioned human skeletal muscle myoblasts (HSMM) myotube medium (Figure 4B).

Treating the MDA-MB-231 cells with the myokine mix of CXCL1, IL10, and CCL4 for 72 h also resulted in a significantly higher *Casp3* mRNA expression than in the untreated cells (*p* < 0.05). The *Casp7* gene expression was also upregulated, although it did not reach statistical significance (Figure 4C).

**Figure 5:**
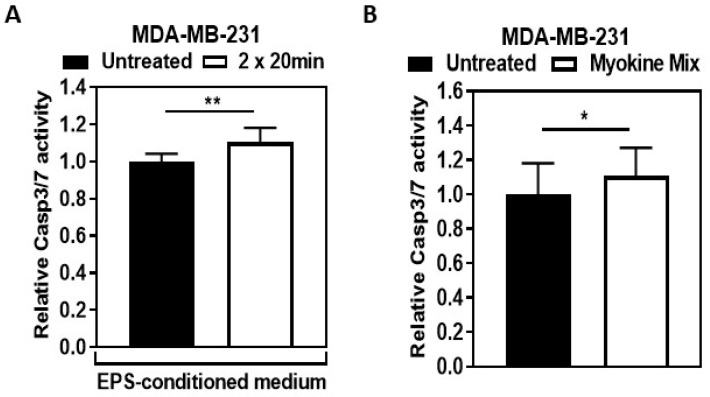
**EPS-conditioned medium and myokine mix induce caspase 3/7 activity in the MDA-MB-231 cell line.** (**A**,**B**). Caspase 3/7 activity was assessed through fluorescent assay using EnzCheck Caspase-3 activity assay kit and standard fluorescein (FITC), with excitation/emission wavelengths of 490 nano (n) metre (m)/570 nm. (**A**). Cells were treated with the EPS-conditioned medium from human myotubes for 48 h (n = 5). (**B**). Myokine mix treatment was performed on the cells for 48 h (n = 7). Graphs display mean (SD) values. Statistical analysis was done using Student’s *t*-test in (**A**,**B**). * *p* < 0.05, ** *p* < 0.01.

Treating MDA-MB-231 cells with EPS-conditioned medium or myokine mix for 48 h also resulted in a significantly elevated induction of Caspase 3/7 activity (Figure 5A: *p* < 0.01, Figure 5B: *p* < 0.05) compared to non-stimulated MDA-MB-231 cells.

## 3. Discussion

Evidence indicates a correlation between higher levels of physical training and a lower incidence of BC [9,10,11,12]. Moreover, increased physical training is associated with improved survival rates among BC patients [13,14]. The positive long-term effects of exercise on BC patients are multifaceted, encompassing risk reduction, an enhanced quality of life, and improved functional outcomes for survivors [15]. These benefits may be associated with various mechanisms, such as metabolic regulation, immunomodulation, anti-inflammatory effects, and hormonal balance [16]. Clinical trials have explored exercise interventions for BC patients, focusing on physical and physiological effects [17,18,19]. Notably, aerobic and resistance exercises have been shown to improve the quality of life in BC patients [20]. Interestingly, the effectiveness of exercise has been demonstrated both during and after BC treatment. However, initiating the exercise routine soon after diagnosis and maintaining it throughout treatment appears to maximize long-term benefits [21]. Nevertheless, optimal intensity and duration of exercise may vary based on individual factors like health status, cancer stage, treatment modalities (e.g., radiotherapy, chemotherapy, or hormonal therapies), dietary habits (including potential nutritional deficiencies), changes in appetite, lifestyle factors, physical activity levels, and sleep quality. Further research should aim to elucidate the interplay between these factors and exercise recommendations, ultimately leading to more personalized and effective interventions for cancer patients. It is important to note that, despite multiple shreds of evidence demonstrating the positive impact of exercise on BC patients, research specifically examining its direct benefits for muscle strength and cancer protection in this population remains limited. Our previous work showed that resistance training increases the expression of certain myokines with anti-tumor properties [4,5]. Nevertheless, the role of exercise-induced myokines in BC remains largely unexplored. To the best of our knowledge, this study is the first to investigate the influence of these specific resistance training-induced myokines both in vivo in patients with BC and in vitro on cancer cell lines. The time-efficient whole-body electromyostimulation (WB-EMS) training demonstrated significant efficacy in increasing muscle mass in BC patients over 12 weeks, compared to the control group without physical training. Notably, fat mass levels remained consistent between both study groups throughout the intervention, probably due to comparable nutritional therapy across all participants. This observation implies that the physical training specifically targeted skeletal muscle growth rather than inducing general fat gain. Remarkably, bioelectrical impedance analysis (BIA) measurements showed muscle growth as early as the fourth week, highlighting the effectiveness of the physical training. Furthermore, improved hydration status following physical training implies improved muscle mass, which has been described as a key indicator of the positive effects of physical training [22]. The increase in muscle mass in BC patients through resistance training significantly enhances their physical strength, which, in turn, improves their physical performance, as evidenced by increased hand grip strength and improved results in the six-minute walking distance test of the trained patients compared to the control group with no training and also within WB-EMS patient group after the 12-week intervention compared to baseline. Furthermore, the significant elevation in creatinine levels, within the normal range, observed in the physical training group aligns with the existing literature, which has established that physical training and increased muscle mass influence this particular blood parameter [23]. Our data concerning the improvement in muscle mass, strength, and overall physical performance are consistent with the results obtained in BC patients after 17 weeks of resistance training. Courneya et alia (et al.) further showed that resistance training also improved self-esteem and chemotherapy completion rate in these patients [24,25].

Our findings extend beyond clinical parameters, demonstrating that serum derived from exercise-trained BC patients who underwent WB-EMS training significantly inhibited the growth of BC cell lines while also increasing apoptosis. Our data align with the previous literature based on in vivo studies. Notably, murine studies have shown that conditioned serum from exercising mice decreases the proliferation and viability of BC cells through exercise-induced factors [10]. We employed electric pulse stimulation (EPS), a valuable in vitro technique that mimics real-life muscle cell responses to electrical stimulation, to further validate these findings. Using this exercise-mimicking model on human myotubes, we confirmed that the conditioned medium harvested from this in vitro stimulation exhibited significant anti-tumor effects on BC cells, similar to our data with serum from trained patients. The results are consistent with previous in vitro research by Davis et al., who also demonstrated anti-tumor properties in human breast cancer cells through exercise-induced myokines derived from rat skeletal muscles [6].

The role of myokines in modulating cancer cell growth and apoptosis has been well-established [26,27]. Among these, CXCL1 (also known as growth-related oncogene 1 alpha or GRO1α) has been described as a significant myokine involved in inflammation [28], and CXCL1 has further been shown to inhibit BC cell proliferation [29]. Similarly, IL10, known as an immunosuppressive cytokine and exercise-induced muscle factor, has been associated with anti-tumorigenesis by downregulating, e.g., vascular endothelial growth factor (VEGF), tumor necrosis factor-α (TNF-α), and nuclear factor ‘kappa-light-chain-enhancer’ in activated B-cell (NF-κB)-mediated pathways [30]. CCL4, also known as macrophage inflammatory protein-1 beta (MIP-1β), functions as a chemotactic agent for various immune cells and has been identified as an exercise-activated muscle factor. While the direct anti-cancer effects of CCL4 have been demonstrated in pancreatic cancer cells [4], its potential impact on BC remains unexplored. In our previous study, we observed that a combination of CXCL1, IL10, and CCL4 significantly impacted pancreatic cancer cells [4]. Extending this line of research to BC, our current findings demonstrate that this myokine mix effectively reduces proliferation and induces cell death in BC cells. These results further support the potential anti-cancer effects of these myokines.

To gain an understanding of the mechanisms underlying cell death, we investigated Caspase 3/7 expression as a marker of apoptosis in cancer. Notably, we observed that the exposure of BC cells to serum derived from patients who underwent resistance training led to an upregulation in Caspase 3/7 expression. Our in vitro exercise model using the electrical pulse-stimulated conditioned medium further confirmed this regulatory effect. Moreover, treating BC cells with a combination of CXCL1, IL10, and CCL4 also increased Caspase 3/7 expression. Our results, therefore, underlined the role of resistance training and myokines in promoting caspase activation in BC cells. Previous studies also investigated the anti-cancer effects of myokines on cancer cells. For instance, in vivo and in vitro studies have demonstrated that oncostatin M and irisin may induce apoptosis in BC cells through caspase activation [10,31]. Thus, there may be several myokines contributing to the inhibition of BC cells through different mechanisms that still have to be identified in further studies.

Our current findings demonstrated that resistance training and myokines trigger a strong anti-tumor response from skeletal muscle, offering significant promise for BC patients. These results align with previous observations in prostate and pancreatic cancers [4,5], suggesting that the benefits of resistance training may be broadly applicable across various cancer types. This observation highlights the need to incorporate resistance training into existing cancer therapies and to develop integrative treatment strategies that provide a non-invasive option for more effective cancer therapy. Furthermore, these findings could help to identify novel therapeutic targets based on myokines and thus provide new strategies in cancer treatment.

## 4. Materials and Methods

Serum samples from patients with BC were examined to investigate the influence of exercise-derived myokines on BC cell proliferation and apoptosis. The patients were randomized into two groups: one received resistance training using the optimized WB-EMS training protocol, and the other represented a control group without exercise therapy [7].

### 4.1. Ethical Approval

The study was carried out according to the principles outlined in the Declaration of Helsinki. The study protocol received approval from the ethical committee of Friedrich-Alexander-University Erlangen-Nürnberg (Registration number 155_13B, 16 July 2013) and is registered at clinicaltrials.gov (NCT02293239). The subjects were informed and signed consent was obtained.

### 4.2. Clinical Study Design, Patient Recruitment, and Resistance Training as an Intervention

Participants were recruited and the study was conducted at the Hector Center for Nutrition, Exercise, and Sports, Department of Medicine 1, Friedrich-Alexander-University Erlangen-Nürnberg, Germany. The study included patients mostly belonging to German nationality, aged 18 years or older, who had been diagnosed with BC and were undergoing anti-cancer therapies, such as chemotherapy. Additionally, participants needed to show a Karnofsky performance index between 60% and 100% to be eligible for the study. The intervention group received resistance training twice weekly for 12 weeks through the WB-EMS physical training protocol with the miha bodytec device (miha bodytec GmbH, Gersthofen, Germany). WB-EMS is a form of resistance training that triggers a noticeable muscle contraction through electrical stimulation via electrodes, thus enabling whole-body strength training. In WB-EMS, low-frequency electrical impulses (<100 Hertz) with a low intensity (<100 milli-Ampere) are administered via electrodes. Electrical stimulation was enabled by bipolar pulses with a frequency of 85 Hertz and a pulse width of 350 microseconds. This resulted in a pattern of intermittent stimulation with a 6 s pulse phase followed by a 4 s rest phase [32]. Each muscle group has its own controller, with which the current intensity is increased to a level that is just below the point of physical discomfort or pain, and the training sessions last 20 min [5]. The training was supervised by professional sports therapists. Both the sports intervention group and the control group received nutritional therapy from professional nutritionists. According to the guidelines, a daily protein intake of 1.0 to 1.2 g per kilogram of body weight was ensured. For this purpose, nutritional counseling was provided at the beginning of the study and every four weeks. Patients with decreased food intake received medical nutritional therapy, e.g., oral nutrition supplements. The patients’ total energy and nutrient intake were assessed, and their individual dietary intake was continuously monitored during the study period. There was no difference in terms of macro- and micronutrients throughout the period of study. Other potential factors, such as the effects of concurrent treatments and the changes in lifestyle of the patients, that could impact the study’s outcomes were also closely monitored and followed up if reported during the patient visits.

**Figure 6:**
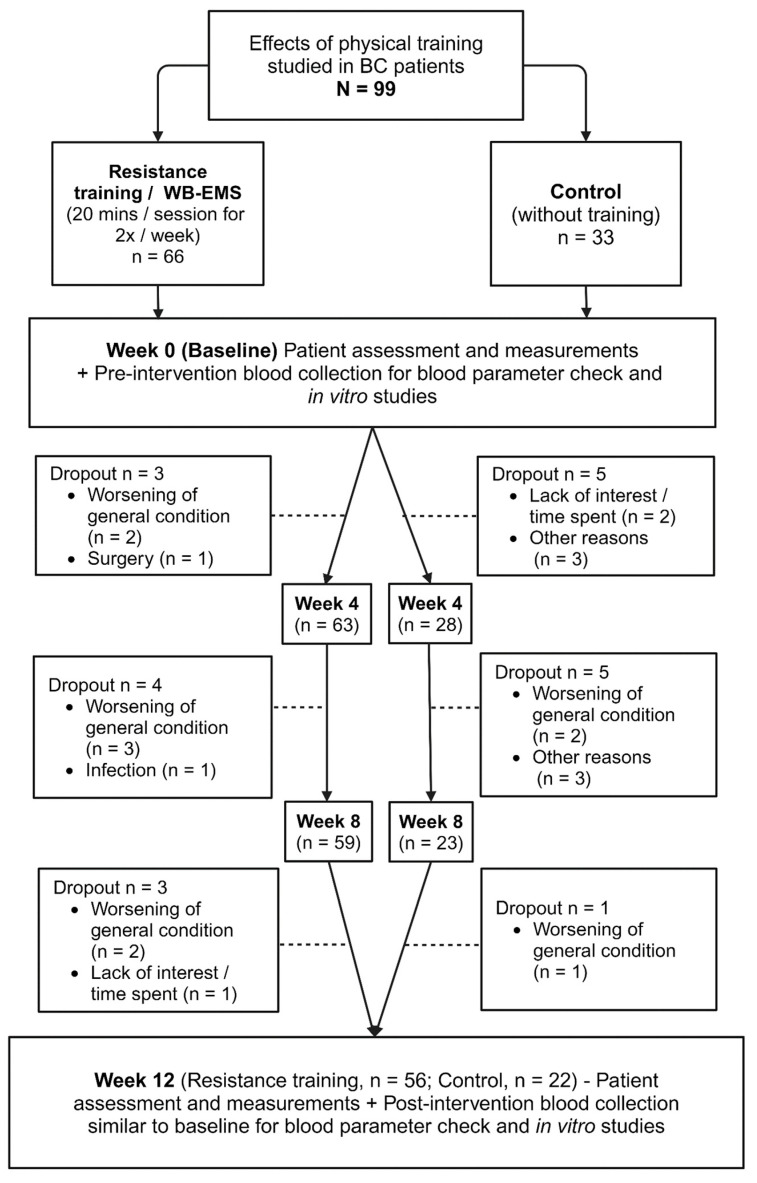
**Study workflow.** The breast cancer (BC) patients were derived from a controlled pilot trial [7] and recruited at the Hector Center for Nutrition, Exercise, and Sports, Department of Medicine 1, Friedrich-Alexander-University Erlangen-Nürnberg. Physical training was conducted over a period of 12 weeks. Initial sample sizes were n = 66 for the resistance training (WB-EMS) group and n = 33 for the non-physically trained control group. By the 12th week of the study period, the final sample sizes were n = 56 in the WB-EMS group and n = 22 in the control group. Dropouts occurred in both groups during the study. Reasons for dropout included worsening general condition, surgery, infection, or other unspecified reasons. This figure was created using BioRender.com (Toronto, Ont. Canada; accessed on 19 August 2024).

At the beginning and during the study, dropouts occurred in both study groups (physical training/WB-EMS, n = 10; controls, n = 11) due to a deterioration in patients’ general condition (WB-EMS, n = 7; controls, n = 3), a loss of interest in the study (WB-EMS, n = 1; controls, n = 2), infection (WB-EMS, n = 1), unplanned surgeries (WB-EMS group, n = 1), and other unspecified reasons (controls, n = 6) (Figure 6).

### 4.3. Patient Physical and Functional Characteristics, Patient Blood Collection, and Measurements

Blood was collected from all patients at the onset of the trial (pre) and 1 h after the last physical training session (post), which was 12 weeks after the start of the trial. The patients’ demographics and disease characteristics, including routine blood parameters, were measured and evaluated at baseline (Table 1) and at the end of the study. Body weight, skeletal muscle mass, fat mass, hydration, and phase angle were determined by bioelectrical impedance analysis (BIA; using Seca Medical body composition analyzer from Seca, Hamburg, Germany). Hand grip strength was measured using a Saehan hand dynamometer (Changwon-si, Republic of Korea).

Venous blood samples were collected and underwent centrifugation. The resulting serum samples were directly analyzed or stored at −80 °C until they were used for in vitro experiments. After excluding the patients who had dropped out from the study, the pre- and post-intervention serum samples were collected from n = 18 control subjects and n = 24 physically trained patients who agreed to provide additional sufficient amounts of blood to perform the in vitro experiments with BC and non-cancer cell lines.

### 4.4. Cell Culture

The MDA-MB-231 cell line (American Type Culture Collection (ATCC), Manassas, VA, United States of America (USA)), derived from BC adenocarcinoma, was grown in Leibovitz’s L-15 medium (Gibco, Thermo-Fisher Scientific, Waltham, MA, USA) supplemented with 10% fetal calf serum (FCS Superior; Merck KGaA, Darmstadt, Germany) and 100 International Unit (IU)/mL penicillin/100 µg/mL streptomycin (Gibco, Thermo-Fisher Scientific, Waltham, MA, USA) at a temperature of 37 °C, 5% CO_2_ in a humidified incubator. The cells (Research resource identifier (RRID): Cellosaurus (CVCL)_0062) were cultured for a maximum of 30 passages. The Michigan Cancer Foundation-7 (MCF7) cell line (ATCC, Manassas, VA, USA), derived from adenocarcinoma breast tissue, was cultivated in Rosewell Park Memorial Institute (RPMI) 1640 medium without phenol red (Gibco, Thermo-Fisher Scientific, Waltham, MA, USA). The culture medium also consisted of 10% FCS Superior and 100 IU/mL penicillin/100 µg/mL streptomycin. The cells (RRID: CVCL_0031) were incubated at 37 °C, 5% carbon dioxide (CO_2_) in a humidified atmosphere, and used for experiments until passage 30. The human embryonic kidney 293T (HEK293T) cells (ATCC, Manassas, VA, USA), derived from the human embryonic kidney, were cultured in Dulbecco’s Modified Eagle Medium (DMEM; from Gibco, Thermo-Fisher Scientific, Waltham, MA, USA) supplemented with 10% FCS Superior and 100 IU/mL penicillin/100 µg/mL streptomycin at 37 °C, 5% CO_2_, humidified atmosphere. The cells (RRID: CVCL 0045) were maintained in culture until 30 passages. The human skeletal muscle myoblasts (HSMMs; from ATCC, Manassas, Virginia, USA) were cultivated in a medium from StemLife Sk LifeFactors Kit (Lifeline Cell Technology, Frederick, MD, USA) that was supplemented with 10% FCS Superior and 100 IU/mL penicillin/100 µg/mL streptomycin at 37 °C, 5% CO_2_ in a humidified atmosphere. There were no traces of mycoplasma in any of the cell cultures.

### 4.5. Electrical Pulse Stimulation (EPS)

Human myotubes were stimulated by EPS to mimic physical training (WB-EMS) and to investigate the effects in vitro. For this procedure, a total of 1.5 × 10^5^/well primary human skeletal muscle myoblasts (HSMMs) were seeded in 6-well plates. Once the HSMMs reached a confluency of 70–80%, they were cultured for 3–4 days with DMEM/Ham F12 (Gibco, Thermo-Fisher Scientific, Waltham, MA, USA) supplemented with 2% Hyclone^TM^ horse serum (GE Healthcare, Chicago, IL, USA) and 100 IU/mL penicillin/100 µg/mL streptomycin (Gibco, Thermo-Fisher Scientific, Waltham, MA, USA) at a temperature of 37 °C. This incubation period allowed the HSMMs to differentiate into myotubes. The effectiveness of myogenic differentiation was assessed by examining the expression of myogenin and the degree of myoblast fusion. EPS was carried out with the differentiated myotubes, and the efficiency of EPS treatment was evaluated according to the previous protocol [5].

### 4.6. Preparation of Myokine Mix and Counteracting Antibody Mix for Exercise-Induced Myokine-Based Assays

Former data from our working group showed that the cytokines CXCL1, IL10, and CCL4/macrophage inflammatory protein-1 beta (MIP-1β) were upregulated in patients with pancreatic cancer (PC) after 12 weeks of resistance training. Since a mixture of these newly identified myokines also inhibited the proliferation of PC cells in vitro, we analyzed the effect of these myokines on BC cells in vitro. A myokine mix was prepared by combining 20 nanograms (ng)/mL of each of the recombinant human cytokines CXCL1, IL10, and MIP-1β (stock concentrations 0.1–1 mg/mL) that were acquired from Peprotech, Cranbury, NJ, USA [4]. To verify the effects of these myokines and their regulatory properties on BC cells, we performed a neutralization assay with corresponding antibodies (Invitrogen, Thermo-Fisher Scientific, Waltham, MA, USA). We combined 1 µg/mL each of anti-IL10 (stock concentration 1 mg/mL), anti-CXCL1 (stock concentration 0.5 mg/mL), and anti-MIP-1β (stock concentration 0.5 mg/mL) to prepare this antibody mix.

### 4.7. Human Cancer Cell Proliferation and Apoptosis Assays

BC cells (MDA-MB-231, MCF7) and non-malignant cells (HEK293T) were seeded in 96-well plates with 3–5 × 10^3^ cells per well or 48-well plates with 2.5–3 × 10^4^ cells per well. After incubating overnight at 37 °C, the cells were deprived of serum (0.1% FCS) for 18–24 h. Next, the cells were stimulated with culture medium containing 10% serum from patients with BC, or with EPS-conditioned medium harvested from differentiated HSMM myotubes (DMEM with 2% FCS plus 10% serum from pooled healthy individual samples), or with recombinant proteins (CXCL1, IL10, MIP-1β; 20 ng/mL each) diluted in DMEM with 2% FCS for 48–96 h at a temperature of 37 °C. The cell proliferation assay, cell counting, and cell apoptosis assay were performed as described earlier [5]. In short, cells were incubated with 10µM 5-bromo-2′-deoxyuridine (BrdU) 18 h before lysis, and cell proliferation was determined by measuring BrdU incorporation according to the instructions of the cell proliferation Enzyme-Linked Immunosorbent Assay (ELISA) kit (Roche Applied Science, Penzberg, Germany). Cell death was examined by checking deoxyribonucleic acid (DNA) fragmentation using the Cell Death Detection ELISA^plus^ (Roche Applied Science) according to the manufacturer’s instructions. The EnzCheck Caspase-3 activity assay kit (Thermo-Fisher, Invitrogen, Waltham, MA, USA) was utilized to detect the caspase-3/7 (Casp3/7) activity-mediated apoptosis, and the experiments were performed using the protocol provided by the manufacturer.

### 4.8. Quantitative Reverse Transcription-Polymerase Chain Reaction (qRT-PCR)

The cultivated cells were treated with QIAzol^®^ reagent (Qiagen, Venlo, The Netherlands) to isolate the total ribonucleic acid (RNA), following the instructions provided by the manufacturer. Subsequently, 0.5–1 μg of the obtained RNA was converted into complementary DNA using the iScript^TM^ synthesis kit (Bio-Rad, Hercules, California, USA). The CFX Connect Real-Time System and CFX Manager 3.1 software (Bio-Rad) were used to perform quantitative RT-PCR (qRT-PCR). The 2^-ΔΔCT^ method was used to calculate the relative messenger RNA expression with hypoxanthine phosphoribosyltransferase 1 (*HPRT1*) as the reference gene.

### 4.9. Primer Sequences

The synthesis of oligonucleotides was performed by Metabion International AG (Planegg, Germany). The following primers were utilized: human *HPRT1* (forward) 5′CCTGGCGTCGTGATTAGTGAT-3′ and (reverse) 5′-AGACGTTCAGTCCTGTCCATAA-3′; human *CASP3* (forward) 5′-ACTGGACTGTGGCATTGAGA-3′ and (reverse) 5′-GCACAAAGCGACTGGATGAA-3′; human *CASP7* (forward) 5′-AGGGACCGAGCTTGATGATG-3′ and (reverse) 5′-GCACAAACCAGGAGCCTCTT-3′ [33].

### 4.10. Statistical Analysis

The study group differences in Table 1 were analyzed using either an independent samples Student’s *t*-test or Mann–Whitney test. Tables and graphs illustrate the means (SD) or standard error of the mean (SEM), and the specific number of samples and experiments are indicated in the legends of the tables and figures. In Figure 1, statistical analysis for body weight, skeletal muscle mass, and hand grip strength was performed by *t*-test (post versus pre, within the WB-EMS group) and Student’s *t*-test (post, between the patient groups), while statistical analysis for fat mass, phase angle, and hydration was performed by Wilcoxon signed-rank test (post versus pre, within the WB-EMS group) and Mann–Whitney test (post, between the patient groups). To analyze the statistical data on BrdU incorporation, DNA fragmentation, and gene expression following the stimulation of the cells with patient serum, we used either a two-tailed *t*-test (to compare post- versus pre-intervention data within a patient group) or Student’s *t*-test (to compare post-intervention data between patient groups). The Wilcoxon signed-rank test was used to analyze the data from cell counting experiments to compare post versus pre-intervention within a patient group, while the Mann–Whitney test was performed to analyze the same between different patient groups, comparing their post-intervention data after stimulating the cells with patient serum. To evaluate the data from the incorporation of BrdU, DNA fragmentation, gene expression, and protein expression (measuring Casp3/7 activity) experiments following stimulation with the EPS-conditioned myotube medium or with a recombinant protein mix, Student’s *t*-test was performed. On the other hand, the Mann–Whitney test was employed to study cell counting data after EPS-medium treatment. The graphs and statistical analysis were performed using GraphPad Prism 9 software (GraphPad Software, San Diego, California, USA; RRID: SCR_002798). *p* < 0.05 was deemed statistically significant.

## 5. Concluding Remarks

We demonstrated that physical training in vivo, especially resistance exercise, significantly benefits BC patients. It not only enhances their physical strength but also stimulates muscle cells to release myokines, which can inhibit the proliferation of breast cancer cell lines ex vivo and trigger their apoptosis. In addition, medium produced in vitro by the EPS of human muscle cells and a combination of recombinant myokines CXCL1, IL10, and CCL4 also reduced the growth rate and induced cell death of breast cancer cells. This particular combination of myokines showed similar results in prostate and pancreatic cancer in previous studies by our group [4,5]. Overall, our data suggest that myokines induced by physical training, thereby stimulating the muscles, have a significant anti-cancer effect, independent of the type of cancer present.

## Figures and Tables

**Table 1:** **Pre-intervention characteristics of the BC patient cohort (total number, n = 99).** The table displays mean ± standard deviation (SD) values. Statistical analysis was conducted using an independent samples *t*-test ^1^ or a Mann–Whitney test ^2^. UICC—Union for International Cancer Control; m—meter; k, kilo; g, gram; m; milli; L, liter; d, deci; µ, micro.

	Breast Cancer (BC)		
	Control (n = 33)	Resistance Training (n = 66)	*p*-Value
**Age** (y)	54.6 ± 12.1	53.2 ± 12.5	0.602 ^1^
**Tumor Stage** (UICC), n (%)			-
I	12 (36.4%)	26 (39.3%)	
II	9 (27.3%)	19 (28.8%)	
III	5 (15.2%)	9 (13.6%)	
IV	7 (21.2%)	12 (18.1%)	
**Oncological Therapy**, n (%)			-
Chemotherapy	24 (72.7%)	29 (43.9%)	
Other therapies	9 (27.3%)	37 (56.1%)	
**Karnofsky Index** (%)	80.3 ± 11.6 (n = 33)	79.2 ± 10.9 (n = 66)	0.655 ^2^
**Six-minute Walking Distance** (m)	561.7 ± 109.7 (n = 32)	560.2 ± 90.5 (n = 61)	0.943 ^2^
**Body Parameters**			
Body Weight (kg)	69.5 ± 15.4 (n = 33)	70.6 ± 12.9 (n = 66)	0.553 ^1^
Weight Loss in the last 3–6 months (%)	2.6 ± 4.3 (n = 33)	2.8 ± 4.8 (n = 66)	0.962 ^2^
Body Mass Index (kg/m^2^)	25.1 ± 5.3 (n = 33)	25.4 ± 4.5 (n = 66)	0.719 ^1^
Skeletal Muscle Mass (kg)	19.7 ± 3.9 (n = 33)	20.1 ± 3.4 (n = 66)	0.574 ^1^
Phase Angle (°)	4.4 ± 0.8 (n = 33)	4.6 ± 0.5 (n = 66)	0.302 ^1^
**Blood Parameters**			
Albumin (g/L)	41.91 ± 2.81 (n = 28)	42.30 ± 3.11 (n = 54)	0.491 ^1^
C-reactive Protein (mg/L)	3.01 ± 2.58 (n = 30)	5.01 ± 8.25 (n = 58)	0.622 ^1^
Creatinine (mg/dl)	0.69 ± 0.10 (n = 31)	0.73 ± 0.13 (n = 60)	0.129 ^1^
Hematocrit (%)	36.65 ± 4.69 (n = 30)	35.74 ± 6.3 (n = 57)	0.487 ^1^
Hemoglobin (g/dl)	15.68 ± 18.83 (n = 30)	12.27 ± 1.54 (n = 57)	0.330 ^1^
Leucocytes (×10^3^/µL)	7.15 ± 4.12 (n = 30)	6.85 ± 7.42 (n = 57)	0.836 ^2^
Erythrocytes (×10^6^/µL)	4.12 ± 0.60 (n = 30)	4.07 ± 0.55 (n = 57)	0.666 ^2^
Thrombocytes (×10^3^/µL)	268.17 ± 77.71 (n =30)	239.11 ± 79.72 (n = 57)	0.107 ^2^

## Data Availability

Data is contained within the article and Supplementary Material.

## Supplementary Materials

The following supporting information can be downloaded at: https://www.mdpi.com/article/10.3390/ijms252111379/s1.

## Abbreviations

BC: breast cancer; WB-EMS, whole-body electromyostimulation; EPS, electric pulse stimulation; PC, pancreatic cancer; CXCL1, C-X-C motif ligand 1; IL10, interleukin 10; CCL4, C-C motif chemokine ligand 4; *Casp3*/CASP3, caspase 3; *Casp7*/CASP7, caspase 7; SD, standard deviation; UICC, union for international cancer control; m, meter; kg, kilo; g, gram; m; milli; l, liter; d, deci; µ, micro; SEM, standard error of the mean; SMM, skeletal muscle mass; PhA, phase angle; FM, Fat Mass; ECW, extracellular water; ICW, intracellular water; MCF7, Michigan Cancer Foundation-7; h, hour(s); Ctrl/Ctl, control group; HEK293T, human embryonic kidney 293T; FCS, fetal calf serum; DNA, deoxyribonucleic acid; BrdU, 5-bromo-20-deoxyuridine; min, minutes; MIP-1β, macrophage inflammatory protein-1 beta; abs, Antibodies; PBS, phosphate-buffered saline; HSMMs, human skeletal muscle myoblasts; RNA, ribonucleic acid; FITC, fluorescein isothiocyanate; n, nano; BIA, bioelectrical impedance analysis; et al.ia, et al.; GRO1α, growth-related oncogene 1 alpha; VEGF, vascular endothelial growth factor; TNF-α, tumor necrosis factor-α; NF-κB, nuclear factor ‘kappa-light-chain-enhancer’ of activated B-cells; ATCC, American-type culture collection; USA, United States of America; IU, international unit; RRID, research resource identifier; CVCL, cellosaurus; RPMI, Rosewell Park Memorial Institute; CO_2_, carbon dioxide; DMEM, Dulbecco’s modified eagle medium; ELISA, enzyme-linked immunosorbent assay; qRT-PCR, quantitative reverse transcription-polymerase chain reaction; HPRT1, hypoxanthine phosphoribosyltransferase 1.
