# Supplementary material for: The Influence of Physical Training on Breast Cancer: The Role of Exercise-Induced Myokines in Regulating Breast Cancer Cell Growth and Survival
Source: Int J Mol Sci. 2024 Oct 23;25(21):11379. doi: 10.3390/ijms252111379 (PMC11547039; doi:10.3390/ijms252111379)
Supplement: Supplementary file 1 [file ijms-25-11379-s001.zip › ijms-3237307-supplementary.pdf]

Supplementary materials section:

Table S1. The differences in body composition and hand grip strength between patients who performed resistance training (WB-EMS) and control patients without training are shown.

	Effect of resistance training (WB-EMS) compared to controls					
	**Week 4**		**Week 8**		**Week 12**	
	**Difference between study groups [95 % CI]**	***p-value***	**Difference between study groups** [95 % CI]	***p-value***	**Difference between study groups** [95 % CI]	***p-value***
Body composition						
SMM difference in [kg]	0.07 [-0.25; 0.39]	0.667	0.28 [-0.06; 0.63]	0.110	0.38 [0.04; 0.73]	**0.030**
Bodyweight difference in [kg]	0.01 [-0.72; 0.75]	0.969	0.22 [-0.57;1.00]	0.589	0.63 [-0.17;1.41]	0.120
FM difference in [%]	0.21 [-0.47; 0.89]	0.550	0.03 [-0.71; 0.77]	0.929	0.01 [-0.73; 0.75]	0.985
PhA difference in [°]	0.01 [-0.10; 0.12]	0.856	0.08 [-0.04; 0.20]	0.208	0.05 [-0.07; 0.17]	0.419
Hydration difference in [%]	-0.68 [-2.22; 0.86]	0.385	-1.03 [-2.69; 0.63]	0.222	-0.68 [-2.34; 0.98]	0.419
Functional status						
Hand grip strength difference in [kg]	1.21 [-0.38; 2.79]	0.134	1.46 [-0.200; 3.11]	0.085	2.83 [1.18; 4.47]	**0.001**

Table S2. The differences in physical functioning (measured by the six-minute walking distance test) and creatinine levels between patients who performed resistance training and control patients without training are shown.

	**Difference between week 12 and week 0 in Controls**		**Difference between week 12 and week 0 in resistance-trained (WB-EMS) patients**		**Effect of resistance training (WB-EMS) compared to controls**	
	**Mean** [95 % CI]	***p-value***	**Mean** [95 % CI]	***p-value***	**Mean** [95 % CI]	***p-value***
Six-minute walking distance difference in [meters]	-30.25 [-77.04; 16.54]	0.191	30.15 [13.38; 46.93]	**0.0007**	60.4 [21.99; 98.82]	**0.0025**
Creatinine difference in [mg/dl]	-0.03 [-0.07; 0.01]	0.110	0.01 [-0.02; 0.04]	0.159	-0.04 [-0.01; 0.09]	**0.041**
